# The difficult legacy of Turing’s wager

**DOI:** 10.1007/s10827-017-0651-y

**Published:** 2017-06-22

**Authors:** Andrew Thwaites, Andrew Soltan, Eric Wieser, Ian Nimmo-Smith

**Affiliations:** 1 University of Cambridge, Cambridge, UK; 2 MRC Cognition and Brain Sciences Unit, Cambridge, UK

**Keywords:** Computational neuroscience, Philosophy of neuroscience, Policy, Alan Turing

## Abstract

Describing the human brain in mathematical terms is an important ambition of neuroscience research, yet the challenges remain considerable. It was Alan Turing, writing in 1950, who first sought to demonstrate how time-consuming such an undertaking would be. Through analogy to the computer program, Turing argued that arriving at a complete mathematical description of the mind would take well over a thousand years. In this opinion piece, we argue that — despite seventy years of progress in the field — his arguments remain both prescient and persuasive.

‘We are not’, Alan Turing once remarked, ‘interested in the fact that the brain has the consistency of cold porridge’ (Turing 1952). For Turing, the key to understanding the brain lay not in mapping its anatomy or measuring its density, but in characterising its behaviour mathematically. His view is one shared by many. Mathematical equations are expressive and unambiguous — and arguably the only language suited to describing so complex an object.

Recent medical innovations are a reminder of the practical value of these equations. Hearing aids and prosthetic limbs exploit algorithms that mimic the equations of the brain and nervous system (Flesher et al. 2016; Moore 2003), as do novel treatments for neurodegenerative disorders such as Parkinson’s, dementia and locked-in syndrome (Chou et al. 2015; Famm et al. 2013; Lebedev and Nicolelis 2006; Vansteensel et al. 2016). Beyond medicine, mathematical characterisations of the brain inform the field of artificial intelligence and the design of novel computer components (Hassabis 2012; The Economist 2013). As the demand for such technology increases, so too does the need to characterise ever more of the brain in mathematical terms.

Policymakers have reflected these aspirations in their funding decisions. In 2013, the European Commission awarded the Human Brain Project [HBP] €1 billion with the explicit aim of constructing an accurate mathematical model of the brain (Markram 2012). Later that year, the US government launched the BRAIN Initiative, aiming to invest $4.5 billion over twelve years towards a study of the brain in which modelling was intended to play a substantial role (National Institutes of Health 2014). China and Japan have since announced projects with similar aims (Grillner et al. 2016), and there appears to be every reason to hope that detailed mathematical models of the healthy and diseased brain may one day become viable and useful propositions.

Yet the difficulties involved are substantial. Turing himself, a pioneer of computational theory, viewed the enterprise with blunt scepticism and sought to highlight the challenges involved with a practical illustration. He started by writing a short computer program on his departmental workstation at the University of Manchester. This program accepted a single number, performed a series of unspecified calculations on it, and returned a second number. It would be extremely difficult, Turing argued, for anyone to guess these calculations from the input and output numbers alone. Determining the calculations taking place in the brain, he reasoned, must be harder still: not only does the brain accept tens-of-thousands of inputs from sensory receptors around the body, but the calculations these inputs undergo are far more complicated than anything written by a single programmer. Turing underscored his argument with a wager: that it would take an investigator at least a thousand years to guess the full set of calculations his Manchester program employed. Guessing the full set of calculations taking place in the brain, he noted, would appear prohibitively time-consuming (Turing 1950).

If characterising the brain’s equations is so difficult, what accounts for the recent surge in investment? There are two reasons. The first is that researchers are now able to measure physiological properties inside the brain, including blood oxygenation levels and action-potential spike rates (Ulmer and Jansen 2010; Smith et al. 1985). Turing’s wager demonstrates why this development makes guessing the calculations of the brain easier: if an investigator was allowed to measure the electrical charge of different components inside the Manchester computer while it was running, this would constrain the number of equations that the investigator need consider. The same logic holds true of characterising the brain.

The second development is that modern supercomputers are now able to take on the burden of testing equations themselves. In 1950, an engineer trying to establish the program running in Turing’s computer would have to hypothesise each possible sequence of calculations, use these to predict what the output would be, and then test if these predictions corresponded with the observed output, repeating the process until they found the correct calculation. Today, modern supercomputers are able to test thousands of predictions a second, vastly speeding up the search.

As neuroimaging resolutions and supercomputing speeds have improved, the number of established brain-related equations has grown. Neuroscientists have employed a wide variety of equation-finding strategies, each of which makes use of different configurations of brain measurements and biological assumptions [see (Eliasmith and Trujillo 2014), (de Garis et al. 2010) for review]. The apparent pace of these advances has led some commentators to argue that a complete catalogue of the brain’s equations may be in reach, perhaps within the next fifty years (Kurzweil 2012; Markram 2012).

This is unfortunate, because such optimism is almost certainly misplaced. This is largely because the wager is less influenced by technological advances than it first appears. Generating hypothetical equations, for example, is a considerable bottleneck. Many things the mind does — such as face recognition or language comprehension — are extremely difficult to describe mathematically, and few equations that claim to model such processes have been proposed. Although supercomputers can be programmed to automatically generate, and subsequently test, thousands of equations at a time in an investigatory or heuristic manner (a process known as ‘machine learning’), this is of limited use when the processes they model are intrinsically complicated. Novel equations may come from the emerging fields of artificial intelligence and deep-learning, but the task here is onerous: three of the most requested search terms in the *Kymata Atlas* (a database of equations maintained by the University of Cambridge) are ‘morality’, ‘consciousness’ and ‘ego’ — features of human cognition that no one has yet been able to capture in an equation.^1^

More challenging, however, is the original difficulty the wager aimed to emphasise: that the number of possible equations that an investigator would need to assess in order to fully characterise the brain is unimaginably vast. To determine the calculations behind the Manchester program, the investigator would potentially need to test more candidate equations than there are atoms in the observable universe; to do the same for all — or even part of — the human brain would require the testing of higher numbers still. It remains a stubborn truism that, impressive as recent neuroscience advances have been, they have revealed only a very small proportion of all possible equations taking place. Neither improvements in neuroimaging resolution, nor the current exponential acceleration in computing power, are likely to increase this proportion substantially in the foreseeable future.

These stumbling blocks do not indicate that attempts at brain modelling should be abandoned; after all, the wager does not argue that mathematical modelling of the brain is impossible — only that it is time-consuming. With this in mind, which approaches are likely to be most effective? Aside from the extensive use of supercomputing, two stand out. First are those approaches that restrict their scrutiny to modelling processes related to specific technological applications or clinical conditions, which will, by their nature, be more tractable than mapping the entire brain. Second are those approaches that aim to establish the basic underlying principles of cortical function, in the hope that this will reduce the number of possibilities when determining equations of a more complex nature. Such criteria increasingly influence funding decisions: both the BRAIN and Chinese initiatives favour approaches that demonstrate clinical or applied objectives, or that attempt to reduce the equation search space through research into basic biological principles (National Institutes of Health 2014; Grillner et al. 2016). The European HBP, by contrast, with its explicit aspirations of simulating a complete model of the brain, was required to scale back these ambitions after widespread criticism from the scientific community (Marquardt 2015). This preference for the practical over the comprehensive should be welcomed: given the lack of constraints over what the totality of possible equations in the brain might be, it makes no sense for today’s neuroscience researcher, as with Turing’s investigator, to spend time and resources reaching too far into the distance when useful equations are closer at hand.

Underlying Turing’s wager is a plea for realism. So much has been learnt about the brain over the last twenty years that it is tempting to think that simply offering the field of neuroscience more resources will remove the limitations of the wager and the workings of the brain will be laid bare. This is a mistake. In the endless search for the mathematical basis of the mind, Turing reflected that there was only one truth we can be sure of: ‘We certainly know of no circumstances under which we could say “We have searched enough.”’ (Turing 1950).
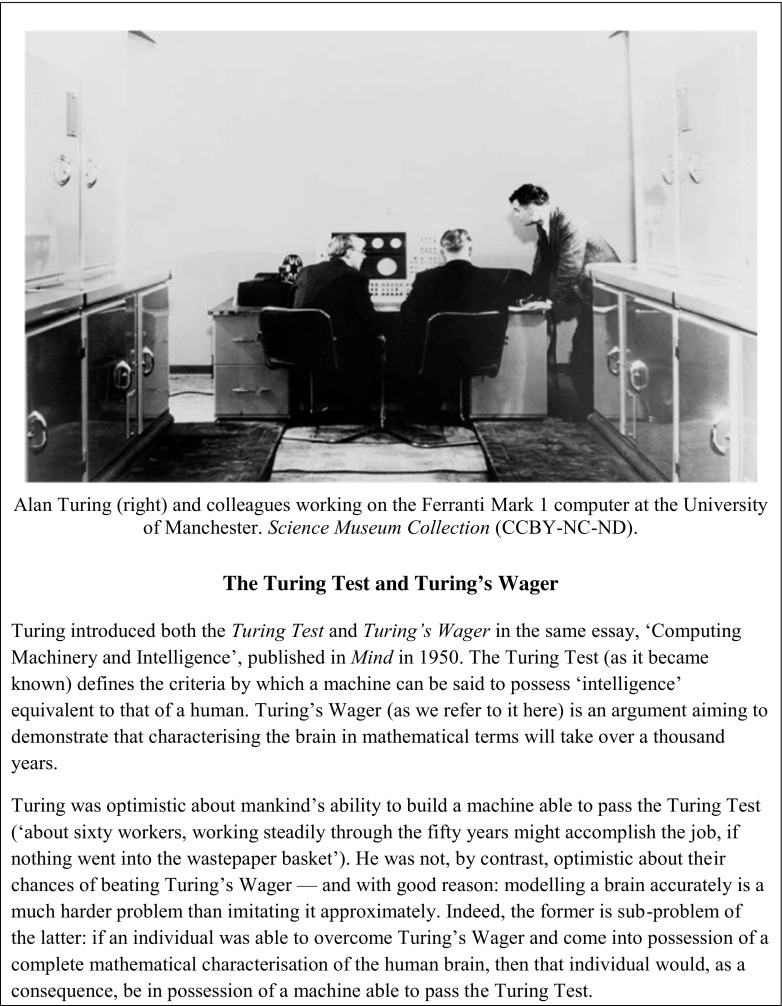


## Footnotes

1 The availability of such equations is not clear cut. *Cf.*, for instance, Tononi and colleagues, who provide what they believe to be plausible mathematical characterizations of consciousness (Tononi 2004; Oizumi et al. 2014), and their critics, who believe these characterizations to be untestable, and therefore unscientific (see Cerullo (2015) for overview).
